# Helical Growth of Aluminum Nitride: New Insights into Its Growth Habit from Nanostructures to Single Crystals

**DOI:** 10.1038/srep10087

**Published:** 2015-05-15

**Authors:** Xing-Hong Zhang, Rui-Wen Shao, Lei Jin, Jian-Yu Wang, Kun Zheng, Chao-Liang Zhao, Jie-Cai Han, Bin Chen, Takashi Sekiguchi, Zhi Zhang, Jin Zou, Bo Song

**Affiliations:** 1 Centre for Composite Materials, Harbin Institute of Technology, Harbin 150080, China; 2 Institute of Microstructure and Properties of Advanced Materials, Beijing University of Technology, Beijing 100124, China; 3 Shenzhen Graduate School, Harbin Institute of Technology, Shenzhen 518055, China; 4 Nano-Electronics Materials Unit, National Institute for Materials Science, 1-1 Namiki, Tsukuba 305-0044, Japan; 5 Materials Engineering and Centre for Microscopy and Microanalysis, The University of Queensland, St. Lucia, Queensland 4072, Australia; 6 Academy of Fundamental and Interdisciplinary Sciences, Harbin Institute of Technology, Harbin 150080, China

## Abstract

By understanding the growth mechanism of nanomaterials, the morphological features of nanostructures can be rationally controlled, thereby achieving the desired physical properties for specific applications. Herein, the growth habits of aluminum nitride (AlN) nanostructures and single crystals synthesized by an ultrahigh-temperature, catalyst-free, physical vapor transport process were investigated by transmission electron microscopy. The detailed structural characterizations strongly suggested that the growth of AlN nanostructures including AlN nanowires and nanohelixes follow a sequential and periodic rotation in the growth direction, which is independent of the size and shape of the material. Based on these experimental observations, an helical growth mechanism that may originate from the coeffect of the polar-surface and dislocation-driven growth is proposed, which offers a new insight into the related growth kinetics of low-dimensional AlN structures and will enable the rational design and synthesis of novel AlN nanostructures. Further, with the increase of temperature, the growth process of AlN grains followed the helical growth model.

The elucidation of the growth mechanism of nanomaterials is one of the most critical topics in nanoscience community, because it is the cornerstone of materials research and applications. Diverse well-known and extensively investigated nanomaterials with simple morphologies such as nanoparticles (NPs), nanorods (NRs), nanocubes (NCs), and nanotubes (NTs) have been synthesized using two general approaches: bottom-up (growth) and top-down (decomposition) by template-assisted and template-free methods^1^. Recently, these anisotropic nanostructures have demonstrated novel physical features and promising utility in nanoelectronics, nanophotonics, solar energy conversion, and electrochemical energy storage, which are strongly dependent on their morphologies and geometries^2^,^3^,^4^,^5^. Thus, it is important to understand the anisotropic growth mechanism of nanomaterials so that the desired nanostructures for specific applications can be synthesized via a rational and well-controlled synthesis strategy. For example, aluminum nitride (AlN), an important III–V semiconductor (a band gap of 6.2 eV), which is an attractive material because of its potential applications in UV-light-emitting diodes (UV-LEDs)^6^, UV dosimetry^7^, field emission devices^8^, and patterned flat panel display^9^. Therefore, novel one-dimensional (1D) AlN nanostructures are highly in demand. Until now, the available 1D AlN nanostructures are limited to nanorings, nanotubes, and nanowires (NWs)^10^,^11^,^12^,^13^,^14^,^15^,^16^,^17^, even though several mechanisms including the electrostatic polar charge model^10^, mismatch between two single-crystal components^11^, and screw dislocation-driven growth model^18^ have been proposed. Now, there is great demand for more structurally complex AlN nanomaterials because the shapes of nanomaterials evidently affect their chemical and physical properties. So far, it remains a challenge to synthesize new 1D AlN nanostructured morphologies such as nanosprings and nanohelixes. Furthermore, the elucidation of the growth mechanism of AlN nanosprings and nanohelixes would be of great interest because of their unique periodic and elastic properties resulting in structural flexibility, thus providing additional opportunities for nanoengineering. Herein, we performed a detailed investigation on the synthesis of AlN nanostructures via an ultrahigh-temperature, catalyst-free, physical vapor transport process. To the best of our knowledge, single-crystal AlN nanohelixes were synthesized for the first time. Moreover, we found that the growth of diverse AlN NWs and nanohelixes with different morphological features can be well explained by the helical growth model, which may result from the coeffect of polar-surface and dislocation-driven growth. Further, with the increase of temperature, this model fitted well with the growth of AlN grains. Based on these experimental observations and theoretical model analysis, the helical growth mechanism can be attributed to the characteristics of AlN itself. This study offers a new insight into the related growth kinetics of the wurtzite (WZ) semiconductors, particularly in the case of AlN. The AlN nanohelixes also add a new member to the growing family of 1D NWs.

## Results

**Figure 1:**
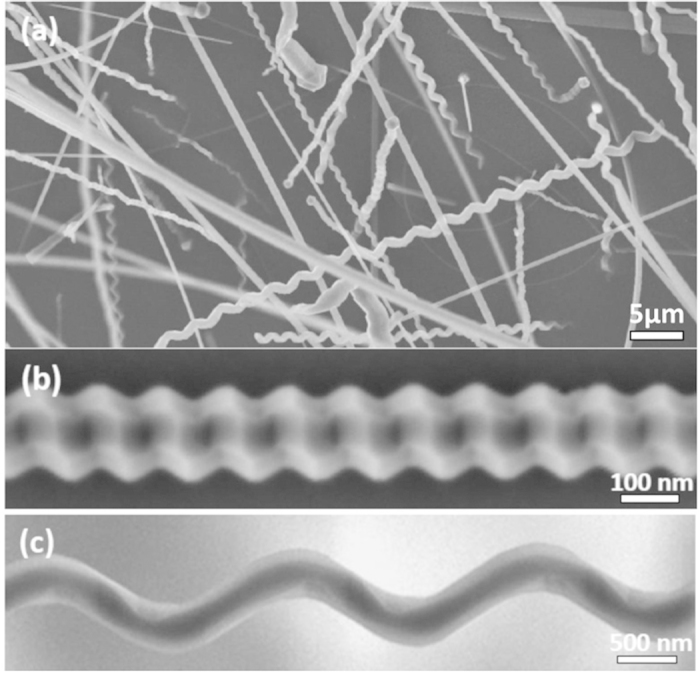
SEM morphologies of AlN nanostructures. (**a**) Low-magnification SEM image showing the general morphology. (**b**) SEM image of the individual AlN NWs. (**c**) SEM image of the individual AlN nanohelix.

The as-prepared products were first examined by scanning electron microscopy (SEM). Figure 1a shows the general morphology of AlN products synthesized at 1800 °C. The diameters of the as-obtained AlN nanostructures ranged from ~100 to 500 nm. The lengths are usually several hundreds of micrometers (μm), and some can approach the millimeter (mm) scale (Fig. S1). In this study, no metal catalyst was used, and no particles were detected at the end of the nanostructures. Therefore, the growth mechanism of the AlN nanostructures can be explained based on a self-catalytic process on the substrate with a vapor–solid (VS) growth mechanism^19^. The X-ray diffraction (XRD) pattern confirms the high crystallinity of the as-prepared products, corresponding to the well-indexed WZ crystal structure of AlN (*a* = 0.3114 nm and *c* = 0.4981 nm, ICDD-PDF-4 + No. 00-025-1133, Fig. S2a). As shown in Fig. S2b, the Raman peaks located at ~612.1, 657.7, and 670.5 cm^−1^ can be attributed to the *A*_1_(TO), *E*_2_(high), and *E*_1_(TO) vibration modes, respectively, which are also the characteristics of WZ AlN nanostructures as reported previously^20^. Semi-quantitative energy-dispersive X-ray spectroscopy (EDS) with a detection limit of 1-2 at.% for the individual AlN NW showed mainly Al and N at a nominal atomic ratio of 1:1 without any other impurities (Fig. S3). Figure 1a also shows that the as-prepared products contain a large number of helical-structured AlN NWs. Helixes consisting of both right- and left-handed chiralities were observed for ~50% distribution. Note that the AlN nanostructures with well-controlled morphologies were found in the central part of the TaC crucible lid, while at the rim of the lid, some irregular AlN nanostructures (Fig. S4) were observed probably owing to the effect of an inhomogeneous temperature field^20^. In general, the ratio of the AlN NWs and nanohelixes to the total number of nanostructures was >80%.

To obtain the morphological features of the as-prepared nanostructures more clearly, the products were sonicated in ethanol for 10 s to disperse and then dropped onto a Si (100) substrate for further high-resolution SEM characterizations. Two main types of undulating surfaces with an orderly texture were observed as shown in Fig. 1b,c. Besides the ordered structures, some undulating surfaces with disordered structures were also observed as shown in Fig. S 4a–c, in which the NW in Fig. S 4a and 4b was flat on the substrate, while the NW in Fig. S 4c lied on the substrate at an angle. In addition, we notice that all the surfaces, regardless of the ordered or disordered structure, show a rough characteristic, raising a question for the formation of such a morphology? The structural information of the as-prepared AlN NWs was further investigated by TEM and model analysis, and the results are reported in the following sections.

**Figure 2:**
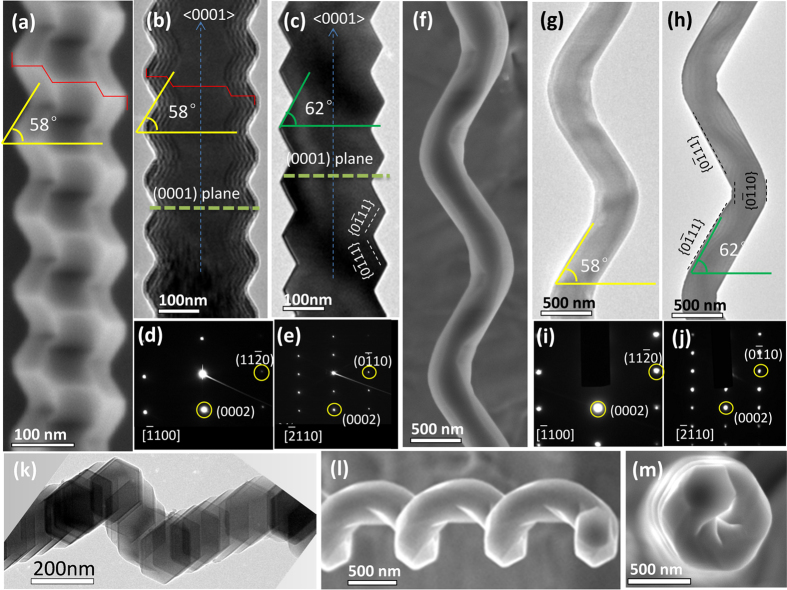
Morphologies and structural characterizations by SEM and TEM. (**a**) SEM image of the AlN NW with a zigzag morphology, showing the stereoscopic feeling. (**b**) and (**c**) bright-field TEM images projected from the [−1100] and [−2110] directions, respectively. (**d**) and (**e**) are the SAEDPs corresponding to (**b**) and (**c**) respectively. (**f**) SEM image of an AlN nanohelix. (**g**) and (**h**) TEM images of an AlN nanohelix projected from the [−1100] and [−2110] directions, respectively. (**i**) and (**j**) are the SAEDPs corresponding to (**g**) and (**h**) respectively. (**k**) TEM image of another helical AlN NW, which is nonperpendicular to the axis of NW. (**l**) SEM image of the AlN nanohelix from the nonperpendicular direction. (**m**) SEM image of the AlN nanohelix from the top view.

To better understand these rough structures, Fig. 2 shows the structural details of these two different morphological features (see Fig. 1b,c) by SEM. Figure 2a shows the SEM image of a NW lying on the Si (100) substrate. Figure 2b,c show the bright-field TEM images of an individual AlN NW with similar morphologies viewed along different axis directions. Figure 2d,e show the selected area electron diffraction patterns (SAEDPs) corresponding to Fig. 2b,c, respectively, revealing the characteristics of a perfect crystal. In Fig. 2d,e, SAEDPs were viewed along the [−1100] and [−2110] directions, respectively. Note that the SAEDPs in Fig. 2e were obtained from Fig. 2d by rotating at an angle of 30°. Based on these almost same features observed from both Fig. 2a,b, the SEM image as shown in Fig. 2a was also viewed along the [−1100] direction. The fringes shown in Fig. 2b are the thickness contours resulting from thickness effects^21^. Herein, we noticed the two angles, 58° and 62°, as shown in Fig. 2b,c, respectively. In fact, one can see clearly that the brim of the NWs was an edge, i.e., this edge meets the axis of <0001> at 32°. Based on the theoretical calculations, the direction of this edge is <−2111>, i.e., it is the actual growth direction of the NW. According to Fig. 2c, it is naturally concluded that the side of the NW is actually a plane, i.e., the measured angle between this plane and {0001} plane is 62°. The theoretical calculation indicates that the angle between {0-111} and {0001} plane was 61.6°; thus, the side plane was confirmed as the {0–111} set. Figure 2f–j show another case with identical characterizations as in Fig. 2a–e, which provided similar information. The only difference between Fig. 2c,h is that there are two {0-110} sets in the side plane of NW as shown in Fig. 2h. In fact, this can be attributed to the very low percentage of this plane in Fig. 2c; therefore, seemingly “sharp” edges were observed, which will be further discussed in the following atomic model section. Similar NWs were tilted at certain angles to observe them, as shown in Fig. 2k,l; then, a clear helical structure was observed. If the nanohelix in Fig. 2l was rotated by an angle of 90°, a distinct six-fold rotation and a clear cross-section with hexagonal features were observed as shown in Fig. 2m. In light of the structural information obtained from the SAEDPs and the corresponding morphologies features, the following information was obtained: 1) a spiral growth along the six equivalent directions, which is <−2111>; 2) the stacking plane of the NW were confirmed as a {0001} plane, i.e., the axis direction is along the <0001> plane; and 3) the side planes are composed by {0-111} and {0-110} sets.

**Figure 3:**
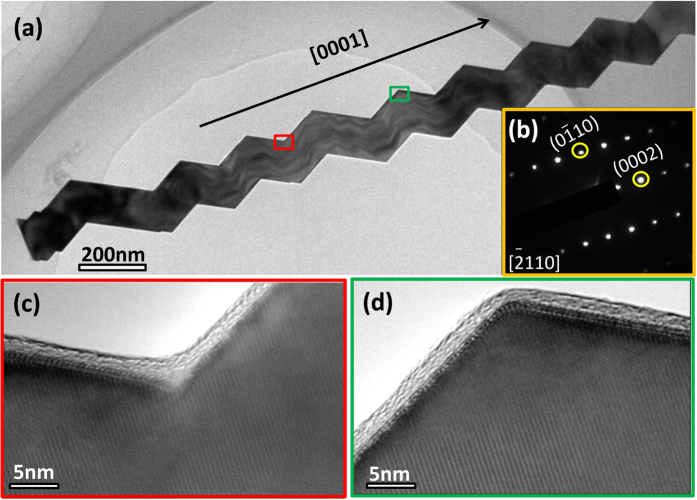
Characterization of an regular AlN NW with zigzag morphology. (**a**) TEM image. (**b**) SAEDPs corresponding to (**a**). (**c**) and (**d**) HRTEM images corresponding to the selected areas as marked by red and green boxes, as shown in (**a**).

**Figure 4:**
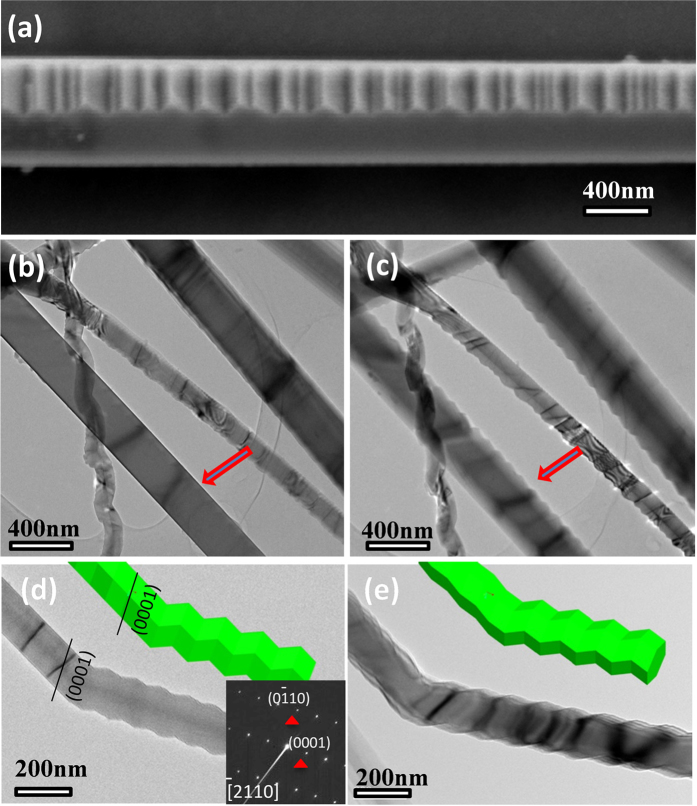
(**a**) SEM image of AlN NW with a smooth surface when viewed from a special direction. (**b**) TEM image of the AlN NW with a typical smooth surface as indicated by the red arrow; (**c**) TEM image recorded when the NW was tilted in TEM, showing a zigzag surface feature; (**d**) TEM image viewed along the [−2110] direction, showing a smooth specific section and another section with a zigzag configuration. Inset shows the corresponding SAEDPs. (**e**) TEM image shows a wave-type surface in the two sections after tilting at a small angle.

**Figure 5:**
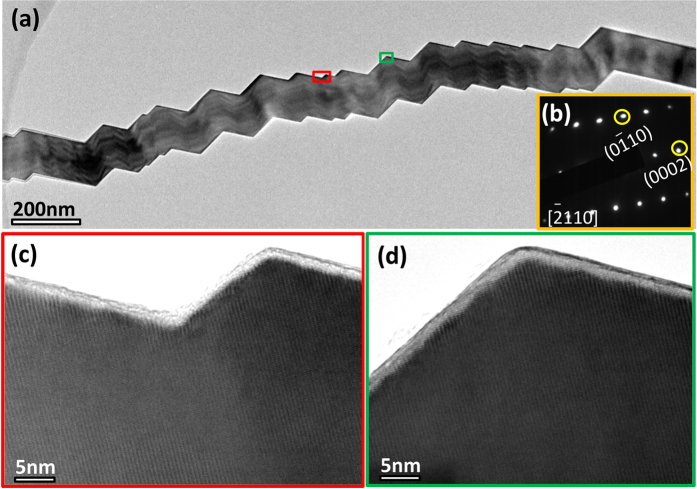
Characterization of irregular AlN NW with zigzag morphology. (**a**) TEM image; (**b**) Corresponding SAEDPs corresponding to (**a**); (**c**) and (**d**) HRTEM images corresponds to the selected areas as marked by red and green box, as shown in (**a**).

Interestingly, at a higher SEM magnification, most of the seemingly straight AlN NWs show a zigzag surface (Fig. S5). Further, besides the aforementioned three types of AlN NWs and nanohelix morphologies as shown in Figs. 1,2, another type of AlN NWs with a smooth surface (Fig. 3) was observed when viewed from a special direction as shown in Fig. 3a. However, these smooth surfaces show a zigzag appearance when they were tilted. As shown in Fig. 3b, an AlN NW with a typical smooth surface as indicated by the red arrow was observed, and a zigzag surface was clearly observed when this NW was tilted in TEM (Fig. 3c). Similar results were obtained for another AlN NW, as shown in Fig. 3d,e, showing a smooth surface when viewed in a specific section, [−2110], and a zigzag surface when viewed from a tilted direction (Fig. 3d). The inset in Fig. 3d shows the corresponding SAEDPs. After tilting at a small angle, a wave-type surface was observed in the two sections (Fig. 3e). These novel helical morphologies raise a basic question that whether it was induced by dislocations or twins? Herein, we present a convincing answer to this question by high-resolution TEM (HRTEM) characterizations. Figure 4a shows the low-magnification TEM image of an individual NW with zigzag morphological features. Figure 4b shows the corresponding SAEDPs, viewed along the [−1100] direction. Figure 4c,d show the HRTEM images corresponding to the high and low spots as marked by a red and green box, respectively. A clear lattice image without any stacking faults and twins was observed, revealing its perfect single-crystal essence. Moreover, similar results were obtained at the randomly selected sites, and we would like to point out that no trace of stacking faults or twins were detected in all the examined AlN nanostructures with regular morphological features (50 out of 50, 100%). One may suspect that the stacking faults or twins may exist in the AlN nanostructures with irregular morphological features. We performed HRTEM characterizations on the seemingly irregular NWs with zigzag morphologies; unexpectedly, all of them (42 out of 42, 100%) exhibited an excellent crystalline quality without the presence of any stacking faults or twins (Fig. 5).

**Figure 6:**
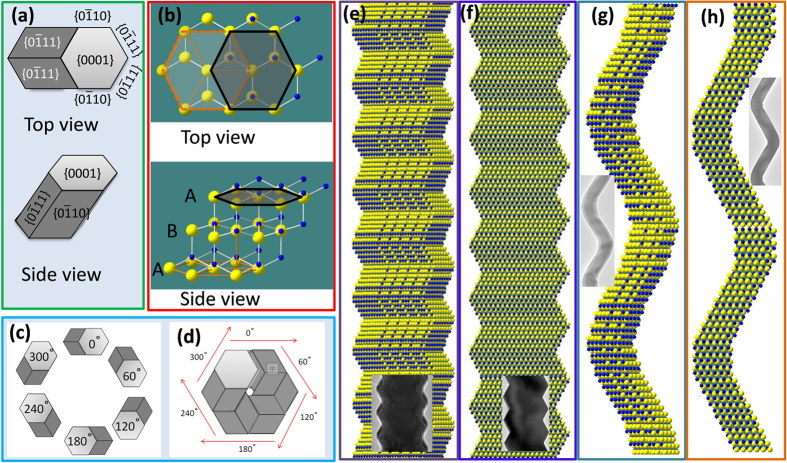
AlN nanostructures and its model. (**a**) Top and side views of the model. (**b**) Top and side views of the atomic model. (**c**) Six identical oblique hexagonal prisms. (**d**) a helical NW constructed from six hexagonal prisms grown in a sequence. (**e**)–(**h**) as-established model for NWs with different morphological features, and the inset shows the corresponding SEM images.

Based on the aforementioned crystal structure characterizations, the following information was obtained: 1) six helical structures; 2) single crystal; 3) the section is a hexagon, and the stacking plane is <0001>. The growth direction is along the <−2111> plane with the side planes, {0–111} and {0–110} sets. Thus, a corresponding model was built as shown in Fig. 6. Figure 6a shows the top and side views of the model. Clearly, the cross-section is a hexagon. This model is composed by two (top and bottom) surfaces of {0001} plane, four equivalent side planes of {0–111}, and two equivalent side planes of {0–110} set. The growth direction is along the six equivalent directions of <−2111> plane. Figure 6b shows the top and side views of the atomic model. It is well known that the hexagonal close packing (*hcp*) structure has a stacking order of ABABAB…… (2*H* structure). Similarly, the as-established atomic model is also an *hcp* structure. In contrast, the third layer “A” move along the <1-210> direction by *a*, and thus, an oblique hexagonal prism is formed. In fact, the third layer “A” can move along any of the six equivalent directions of <1-210>, i.e., growth along any one of the six equivalent directions of <−2111> is possible. Then, six identical oblique hexagonal prisms were obtained as shown in Fig. 6c. If the six hexagonal prisms grow in a sequence, the helical NW as shown in Fig. 6d will be constructed as the helix. According to the atomic model, the model for the NWs was built as shown in Fig. 6e–h. Two types of morphological features are presented perfectly by tuning the thickness-to-diameter ratio of the oblique hexagonal prism, consistent well with the as-observed morphological characters in experiment. To investigate the difference between Fig. 6f,h, one can see clearly that the corner in Fig. 6f is very sharp, while that in Fig. 6h is soft with the presence of {0-110} planes. These observations are also consistent with the observed morphological features as shown in Fig. 2. In fact, when the hexagonal prism does not strictly follow the growth mode of six consecutive helixes or the thickness of the adjacent hexagonal prism is inconsistent with each other, NWs with irregular morphological features will be formed (Fig. S4). However, it will not destroy the single-crystal characteristics of the as-prepared NWs. In fact, the NWs as shown in Fig. 3 can also be built using the oblique hexagonal prisms. So far, to a certain extent, it is found that all the as-prepared NWs are AlN nanohelixes in essence.

This novel result will inspire a new wave of inquiry into one problems: what is the driving force for such growth characteristics. During the past decade, the polar-surface-driven model proposed by Wang and coworkers was successfully utilized to explain the formation of the nanohelixes, nanosprings, nanorings of ZnO and ZnS^22^,^23^,^24^,^25^,^26^. Recently, Jin *et al.* showed that the screw-dislocation-driven growth model was a general and versatile mechanism to grow NWs^18^,^26^,^27^,^28^,^29^,^30^,^31^,^32^,^33^. As a possible explanation for the formation mechanism of nanohelixes, we cite the study in Ref. 25where the polarization arises from noncentral symmetric WZ structure. Herein, the crystal structure of WZ AlN can be described schematically as several alternating planes composed of four-fold tetrahedral-coordinated N^3−^ and Al^3+^ ions, stacked alternately along the *c* axis^10^. In this process, the oppositely charged ions produce a positively charged (0001)-Al polar surface and a negatively charged (000ī)-N polar surface, resulting in spontaneous polarization along the *c* axis. Then, the growth of AlN nanohelixes is led by the Al-terminated (0001) or N-terminated (000ī) front surfaces. However, we still cannot absolutely exclude the possible contribution from dislocation-driven^34^; the detailed HRTEM analysis provides convincing evidence against this possibility. In fact, AlN were shown to grow *via* the screw-dislocation-driven mechanism since the 1960s^35^,^36^,^37^. Recently, Wang *et al.* reported that the lattice mismatch along the boundaries can also serve as a driving force for the formation of bicrystal AlN zigzag NWs^11^.Therefore, the growth mechanism for AlN nanohelixes needs further investigation. Moreover, AlN has the largest value of spontaneous polarization reported so far for any binary compounds, and only a factor of 3–5 smaller than the typical ferroelectric perovskites^38^, indicating that only the polar surface could also induce the helical growth behavior. Compared to the intentionally designed experiment to grow ZnO NWs in which the role of dislocations could be verified^27^, true driven mechanism is still unclear in this study. Herein, helical growth behavior may be the result of combined action of both polar surface and dislocation driven mechanisms, despite that the role of the latter one has not been well proved. Nevertheless, this study shows the growth habit of AlN nanostructures and will benefit the growth of AlN single crystals because the growth process of AlN NWs and nanohelixes *via* the physical vapor transport route is close to the growth environment of AlN bulk single crystals.

**Figure 7:**
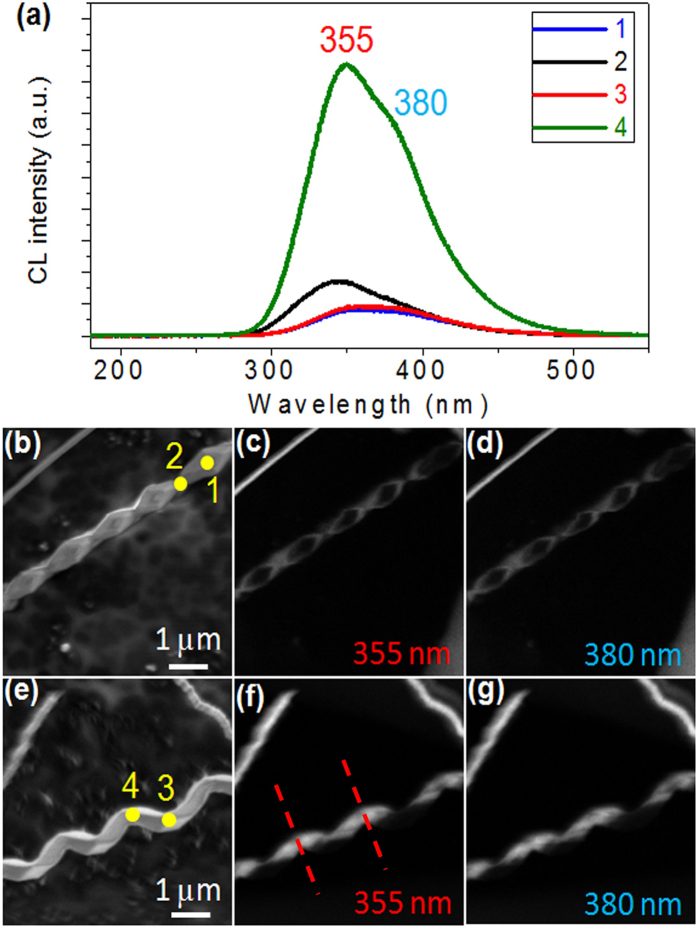
(**a**) CL spectra of AlN nanostructures recorded from the marked positions in (**b**) and (**e**). (**b**)–(**d**), SEM image of AlN NWs and the corresponding CL images recorded at 355 and 380 nm. (**e**)–(**g**), SEM image of the product and the corresponding CL images measured at 355 and 380 nm.

It is speculated that such unique nanostructures may exhibit unusual optical properties, particularly luminescence, which strongly depends on size, orientation, and growth parameters^12^. To investigate the optical properties of as-prepared AlN nanohelixes, cathodoluminescence (CL) spectra were obtained at room temperature under an applied accelerating voltage (*V*_a_) of 5 kV. Figure 7a shows the representative CL spectra recorded from the four sites as marked in Fig. 7b,e. Apparently, AlN nanohelixes exhibit a strong emission centered at 355 nm and a shoulder centered at 380 nm. The emission at 355 nm can be attributed to the recombination of the electrons trapped in oxygen impurities and the holes trapped in the O_N_–V_Al_ complex^39^,^40^,^41^. Moreover, the shoulder located at 380 nm is associated with the oxygen-*DX* center^42^. Although the leakage rate of the furnace is very low, an ultra-high temperature makes it difficult to avoid the incorporation of oxygen impurities completely. Therefore, more or less oxygen atoms may be incorporated into AlN lattices as impurities. To visualize the spatial distribution of the luminescence, CL images were recorded at 355 and 380 nm, as shown in Fig. 7c,d,f,g. By recording SEM images in the same region, as shown in Fig. 7b,e, the luminescence and morphological information of an individual AlN nanohelix could be obtained. CL images show an interesting feature, that is, an alternating “light (sites 2 and 4)” and “dark (sites 1 and 3)” contrast along the growth direction [0001]. The periodic variation in CL intensity may be assigned to the concentration changes in the N vacancies and oxygen-related defects^43^. The reason for such novel features is unclear. In contrast to the periodically “light” and “dark” contrast in AlN nanohelixes, the straight AlN NWs did not exhibit similar features (see the upper left corner in Fig. 7c,d, the upper left and right corners in Fig. 7f,g). In sites 1 and 3, a CL intensity of 355 nm was comparable to that at 380 nm, and both of these peaks were weaker than those at sites 2 and 4. Notably, one period of CL intensity variation, consisting of two “light” and one “dark” as marked by the red dotted line (Fig. 7f), agrees well with one typical building blocks as built by six oblique hexagonal prisms, as shown in Fig. 6e.

**Figure 8:**
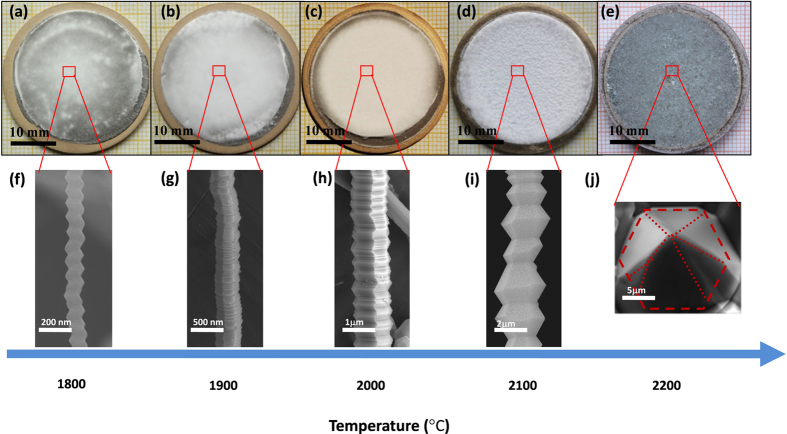
(**a**–**e**) Optical images of the as-prepared AlN samples grown on a TaC crucible lid. From left to right, the temperature increases from 1800 to 2200 °C. (**f**–**j**) SEM images of the as-prepared NWs and grains corresponding to the selected area marked by the red box as shown in the top sample pictures. (**k**–**o**) Growth model corresponding to the upper SEM images from (**f**–**g**).

By far, we have demonstrated that the AlN nanostructures with various morphologies follow the helical growth mechanism. In fact, the clues of similar helical growth with irregular morphological features have been unconsciously displayed in few reports previously; however, the fabrication of AlN nanohelixes and the efforts toward elucidating the growth mechanism have not been performed. Further, it is not sure whether the helical growth mechanism is still valid for AlN bulk single crystal. In this study, the temperature dependence of AlN morphological evolution from nanostructures to bulk single crystals will provide an opportunity to validate the helical growth mechanism. Figure 8a–e show the optical images of the AlN grown on a TaC crucible lid from 1800 to 2200 °C. Figure 8f–j show the corresponding SEM images of the upper samples. Clearly, the regular appearance of the stacking unit (Fig. 8f) gradually transforms into an irregular shape (Fig. 8i). Significantly, these SEM images (Fig. 8f–i) with distinct layer-stacking characteristics provide convincing evidence to demonstrate that the growth of AlN crystals always obey the helical growth model from 1800 to 2100 °C. Finally, a cone body with an irregular appearance was obtained at 2200 °C (Fig. 8j). It is easy to understand such changes according to Eq.(1)^44^:


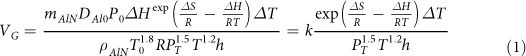


Where *m*_AlN_ is the formula weight, ρ_AlN_ is the density, *P*_0_ is the standard atmospheric pressure, *T*_0_ is 300 K, *D*_Al0_ is the diffusion coefficient of Al vapor at *T*_0_ and under a N_2_ pressure of *P*_0_. *R* is the universal gas constant, *ΔS* and *ΔH* are the sublimation entropy and enthalpy, respectively. *T* is the growth temperature, and *P*_T_ is the total pressure of the system at *T* K. *ΔT* and *h* are the temperature difference and distance between the products and sources, respectively. According to Eq. (1), when the growth was performed at a low temperature, the axial temperature difference is larger than the radial temperature difference, and the *V*_G_ of the {0002} plane is thus larger than that of {10-10} plane. Then, AlN showed a certain preferential growth orientation along the *c* axis^45^,^46^. Moreover, the close-packed {0002} plane probably exhibit a faster growth rate because of the low surface energy^35^,^47^,^48^,^49^. Thus, AlN NWs or nanohelixes with clear helical growth characteristic were obtained. Conversely, when the growth was carried out at a high temperature, the axial temperature difference was smaller than the radial temperature difference. Then, the supersaturation of AlN vapor increases significantly, and the lateral mobility of AlN molecules along the surface of TaC crucible lid also increases remarkably. Then, the *V*_G_ of the (100) plane is comparable or larger than that of (002) plane, and a *m* plane AlN was obtained^50^. Moreover, to meet the requirements of both the lowest system energy and crystallography principle of WZ structure^35^,^47^,^51^,^52^. the zigzag surfaces gradually merge, and a cone-shape AlN with no more than six lateral surfaces was obtained as shown in Fig. 8j.

**Figure 9:**
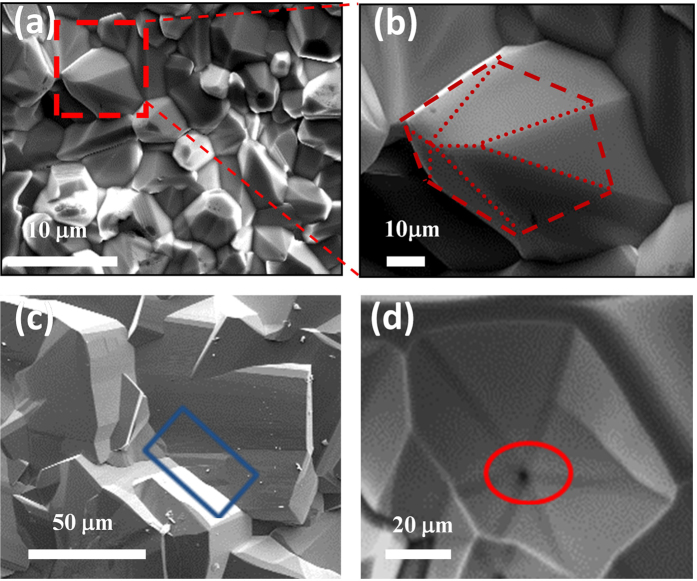
SEM images of the AlN grains obtained at 2300 °C. (**a**) SEM image of the AlN grains with random growth directions. (**b**) Enlarged SEM image of the tilted AlN grains as marked by the red rectangle shown in (**a**). (**c**) SEM image of the AlN grain boundary. (**d**) SEM image of the open-core dislocations (micropipe).

Furthermore, when the temperature increases to 2300 °C, the morphological features change significantly. The appearance of the stacking unit disappears; thus, the fingerprint for the helical growth mechanism could not be detected easily. Although, it is difficult to demonstrate that the helical growth model is still valid at 2300 °C, further investigations into the growth habits will improve the crystallography literacy and benefit us to better understand the growth habits of AlN. Interestingly, some tilted AlN grains were observed (Fig. 9a). Initially, the AlN grain intended to grow along the [0001] direction, the geometric center coincided with the center of gravity. The center of gravity will shift when a large difference exists in the growth rate between the lateral sides, probably resulting from the adsorption of impurity atoms or the local fluctuation in the temperature^45^,^46^,^53^. Finally, the grain leaned to the east, and a tilted grain formed as shown in Fig. 9b. Then, the further growth of the tilted grains will result in the formation of two types of defects when several grains tangle or coalesce together. One is the crystal boundaries as shown in Fig. 9c indicated by a blue rectangle and the other is the open-core dislocations (micropipe) as shown in Fig. 9d denoted by a red ellipse.

## Discussion

Frank *et al.* found similar results in single crystals and films, and then proposed a possible mechanism for such features^54^. It should be note that the formation of AlN micropipe may be promoted by the relaxation of the strain energy induced by the dislocations. Wu *et al.* demonstrated that faceted AlN NTs released the general strain by introducing a finite number of twins, thus lowered the overall thermodynamic free energy^15^. Recently, Jacobs *et al.* suggested the relationship between the axial screw dislocation and the formation of a hollow core^55^. Therefore, the observation of the hollow AlN micropipe suggest the potential contribution from dislocation for the growth of AlN bulk crystals, which require further investigation. Thus, the helical grown mechanism was considered as the feature of AlN, which may originate from the combined effect of polar-surface and dislocation-driven growth mode, even though the latter has not been proved. This study offers a new insight into the related growth kinetics of WZ semiconductors and deeper understanding of the growth mechanism will enable researchers to rationally control the morphologies of other WZ nanostructures.

## Methods

The AlN nanostructures and single crystals were grown by the physical vapor transport method in a RF-heated furnace capable of maintaining temperatures up to 2400 °C with axially symmetric graphite heating elements. A sketch of the experimental configuration of the crucible and growth assembly is shown in Fig. S6. Temperature distribution was adjusted by varying the heating power and heat insulation configuration, and by vertical displacement of the crucible. The temperature was controlled using two pyrometers, one in the center of the crucible lid and the other at the side wall of the crucible. The pyrometers are calibrated using a tungsten-rhenium thermocouple and focused on the crucible surface through two holes in the heat insulation. The preheated AlN polycrystalline was used for synthesizing AlN nanostructures. Approximately 10 g preheated AlN powder was placed in a TaC crucible. Then, TaC crucible was placed inside a graphite crucible. Both these crucibles were placed in the center of the RF-heated furnace. The chamber was first vacuumed to 10^−4^ Pa, and then filled with high purity N_2_ (99.999%) to 10^5^ Pa for three times prior to heating. The N_2_ pressure was maintained at ~6 × 10^4^ Pa. Then, the temperature was raised to 1800–2300 °C from room temperature at a rate of 30–35 °C/min and held at the desired temperature for 40 min. After the reaction was completed, power was turned off, and the furnace was cooled down to room temperature naturally with a flow of 500 sccm N_2_ (99.999%). Herein, the distance between the AlN source material and TaC crucible lid was ~10–15 mm. The estimated temperature gradient between the source and crucible lid was 5–10 °C/mm. The deposited products on the crucible lid were collected for further characterization. Field-emission gun (FEG) scanning electron microscope (JEOL 6500 SEM) and a JEOL FEG HRTEM operating at 200 keV were used to characterize the synthesized materials. The point resolution of the HRTEM was ~0.19 nm. An atomic model of the AlN was compiled using the commercial software CrystalKit, and the HRTEM simulation was performed using the Mactempas. XRD data were collected using a high-resolution X-ray powder diffractometer (Rigaku D/max 2500, CuKα, *λ* = 0.15418 nm). The Raman scattering measurement was performed using a Raman system (JY-HR800) with a 532 nm line from a solid-state laser as the excitation source. Raman spectra were recorded in the back scattering geometry using the non-polarized 532 nm line from a solid-state laser as the excitation source. The resolution of the Raman spectrometer was <1 cm^−1^, and the diameter of the incident light spot was ~1 μm. For the optical measurements, CL spectroscopy was directly performed on the AlN nanohelix samples dispersed on a carbon tape using a field-emission SEM (Hitachi S4300) equipped with a CL system at an accelerating voltage of 5 kV.

## Author Contributions

X.H.Z., L.J. and C.L.Z. designed the experiment and prepared the samples. R.W.S. performed the TEM analysis. Z.Z. performed the SEM analysis. J.Y.W., B.C. and T.S. performed the C.L. analysis and analyzed the data. K.Z. established the atomic model. J.C.H., J.Z., and B.S. analyzed the data, wrote and edited the manuscript. All authors read the paper and commented on the text.

## Additional Information

**How to cite this article**: Zhang, X.-H. *et al*. Helical Growth of Aluminum Nitride: New Insights into Its Growth Habit from Nanostructures to Single Crystals. *Sci. Rep.* **5**, 10087; doi: 10.1038/srep10087 (2015).

## Supplementary Material

Supplementary Information
