# Supplementary material for: Helical Growth of Aluminum Nitride: New Insights into Its Growth Habit from Nanostructures to Single Crystals
Source: Sci Rep. 2015 May 15;5:10087. doi: 10.1038/srep10087 (PMC4432374; doi:10.1038/srep10087)
Supplement: Supplementary Information [file srep10087-s1.pdf]

Supporting Information

Helical Growth of Aluminum Nitride: New Insights into Its Growth Habit from Nanostructures to Single Crystals

Xing-Hong Zhang,[1] Rui-Wen Shao,[2] Lei Jin,[3] Jian-Yu Wang,[4] Kun Zheng,*[,2,5] Chao-Liang Zhao,[1] Jie-Cai Han,[1] Bin Chen,[4] Takashi Sekiguchi,[4] Zhi Zhang, [5] Jin Zou*[,5], and Bo Song*[1,6]

[1]Centre for Composite Materials, Harbin Institute of Technology, Harbin 150080, China

[2]Institute of Microstructure and Properties of Advanced Materials, Beijing University of Technology, Beijing 100124, China

[3]Shenzhen Graduate School, Harbin Institute of Technology, Shenzhen 518055, China

[4]Nano-Electronics Materials Unit, National Institute for Materials Science, 1-1 Namiki, Tsukuba 305-0044, Japan

[5]Materials Engineering and Centre for Microscopy and Microanalysis, The University of Queensland, St. Lucia, Queensland 4072, Australia

[6]Academy of Fundamental and Interdisciplinary Sciences, Harbin Institute of Technology, Harbin 150080, China

* Correspondence to songbo@hit.edu.cn (B. S.), kunzheng@bjut.edu.cn (K. Z.), j.zou@uq.edu.au (J.Z.)

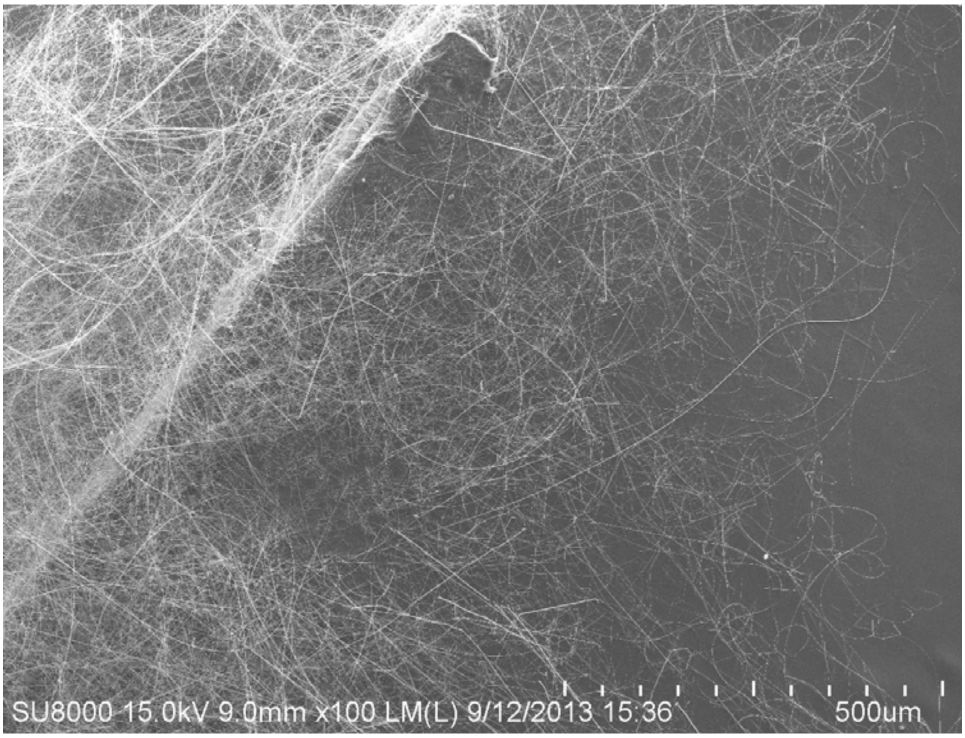

Figure S1 SEM image of the as-prepared product. The as-synthesized materials consist mainly of nanostructures up toseveral hundreds of micrometers long and some can approach the millimeter scale.

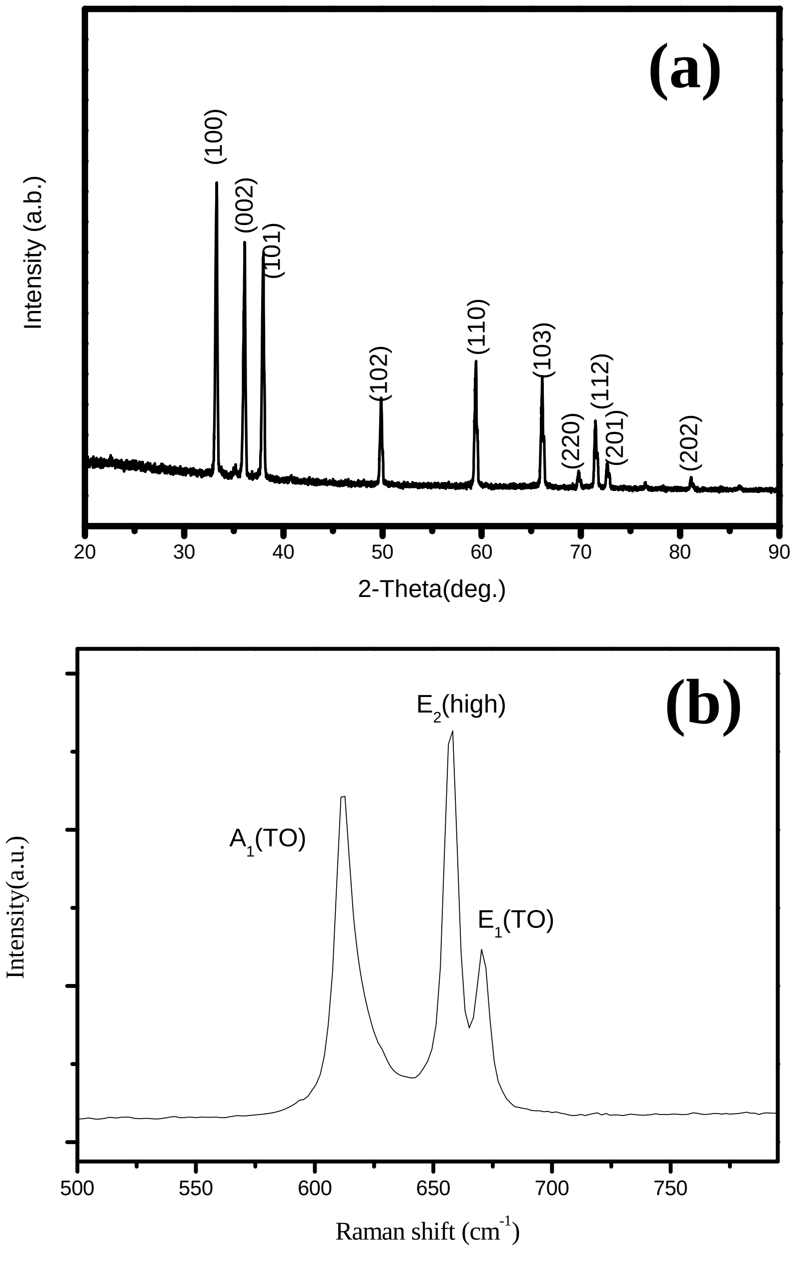

Figure S2 (a)X-ray diffraction pattern of the as-prepared products.(b) Raman scattering spectrum of the as-prepared products.

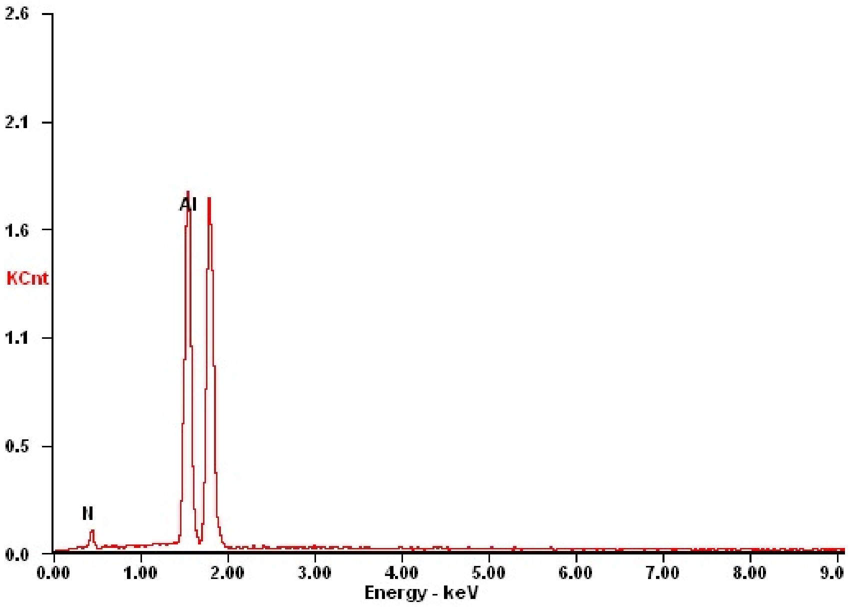

Figure S3 Energy-dispersive X-ray (EDS) analysis of an individual NW on Si substrate. The Al: N ratio is estimated to be 1:1. The Si signal is mainly adventitious and does not vary from sample to sample.

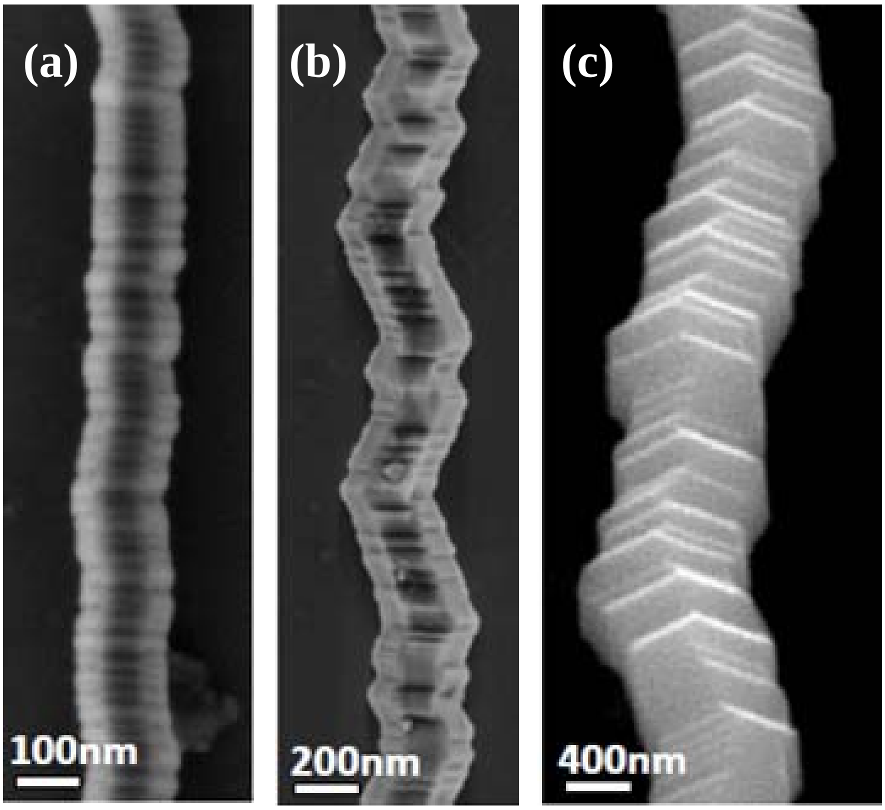

Figure S4(a-c) SEM morphologies of three types of nanohelixes with irregular morphologies features.

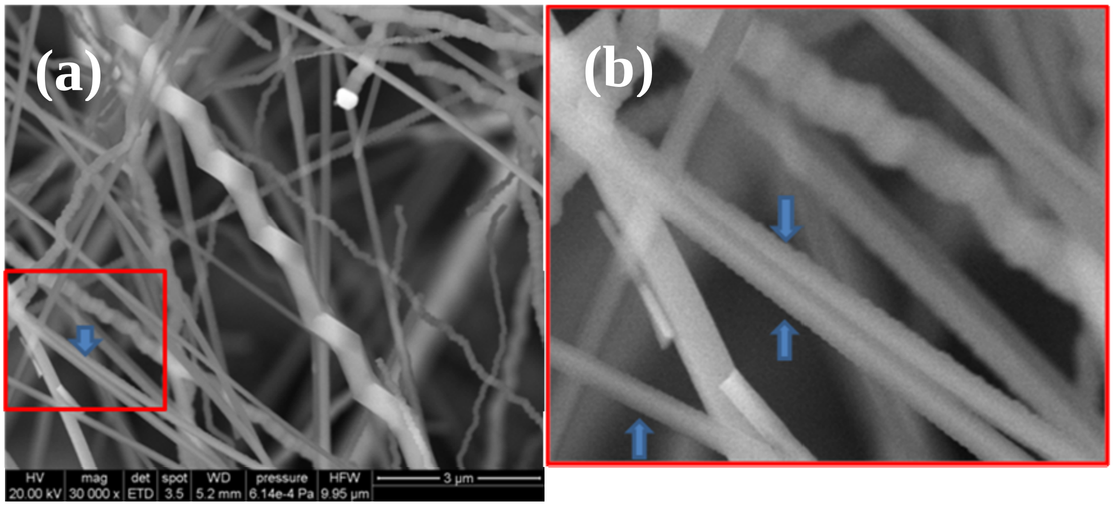

Figure S5 (a) SEM image of one type AlN NWs with the seemingly straight morphologies as indicated by the blue arrow. (b) the magnified SEM image as shown in (a) indicated by the red box.

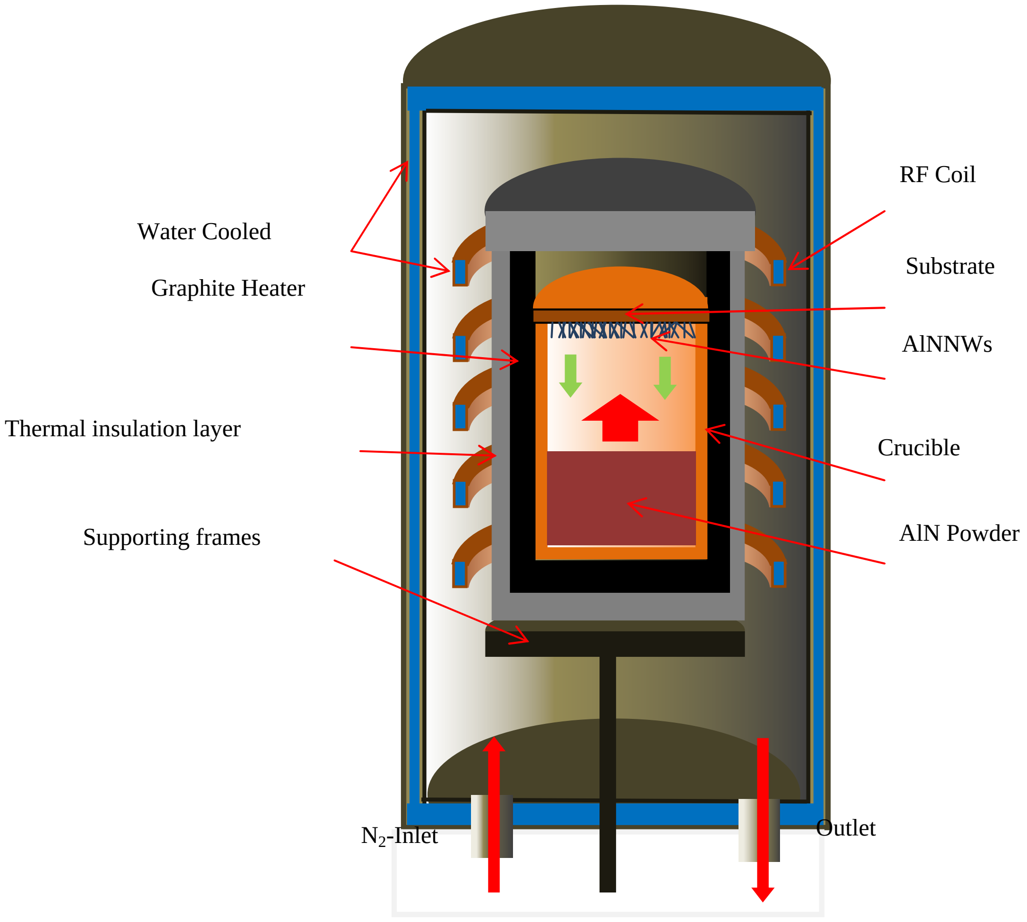

Figure S6 Schematic of the typical PVT sublimation configuration
